# MIRO GTPases in Mitochondrial Transport, Homeostasis and Pathology

**DOI:** 10.3390/cells5010001

**Published:** 2015-12-31

**Authors:** Bor Luen Tang

**Affiliations:** 1 Department of Biochemistry, Yong Loo Lin School of Medicine, National University of Singapore, MD7, 8 Medical Drive, Singapore 117597, Singapore; bchtbl@nus.edu.sg; Tel.: +65-6516-1040; Fax: +65-6779-1452; 2 NUS Graduate School for Integrative Sciences and Engineering, National University of Singapore, 28 Medical Drive, Singapore 117456, Singapore

**Keywords:** MIRO, mitochondria, mitochondrial transport, Milton, small GTPases

## Abstract

The evolutionarily-conserved mitochondrial Rho (MIRO) small GTPase is a Ras superfamily member with three unique features. It has two GTPase domains instead of the one found in other small GTPases, and it also has two EF hand calcium binding domains, which allow Ca^2+^-dependent modulation of its activity and functions. Importantly, it is specifically associated with the mitochondria and via a hydrophobic transmembrane domain, rather than a lipid-based anchor more commonly found in other small GTPases. At the mitochondria, MIRO regulates mitochondrial homeostasis and turnover. In metazoans, MIRO regulates mitochondrial transport and organization at cellular extensions, such as axons, and, in some cases, intercellular transport of the organelle through tunneling nanotubes. Recent findings have revealed a myriad of molecules that are associated with MIRO, particularly the kinesin adaptor Milton/TRAK, mitofusin, PINK1 and Parkin, as well as the endoplasmic reticulum-mitochondria encounter structure (ERMES) complex. The mechanistic aspects of the roles of MIRO and its interactors in mitochondrial homeostasis and transport are gradually being revealed. On the other hand, MIRO is also increasingly associated with neurodegenerative diseases that have roots in mitochondrial dysfunction. In this review, I discuss what is currently known about the cellular physiology and pathophysiology of MIRO functions.

## 1. Introduction

The mitochondrion is an endosymbiont-derived organelle [1] with multiple key roles in eukaryotic energy metabolism and cell survival. It is perhaps best known to students of cell biology as the compartment that houses the respiratory electron transport chain (and the associated processes of oxidative phosphorylation and the generation of reactive oxygen species), as well as key regulators of programmed cell death (such as the apoptosis-inducing cytochrome c and apoptosis inducing factor (AIF)). The mitochondrion is also the site of the tricarboxylic acid (TCA) cycle and fattyacid β–oxidation, and it serves to buffer and sequester intracellular calcium [2,3]. Mitochondria are highly dynamic organelles in terms of morphology and cellular distribution and constantly undergo processes of fusion, fission and cytoskeleton-dependent transport. The latter process is particularly important for cell types and subcellular locations where ATP is acutely required and where adequate maintenance of cytosolic calcium levels is critical, such as the synaptic termini of neurons [4]. Impaired mitochondrial function and homeostasis therefore underlie many neurodegenerative and metabolic disorders [5].

The Ras superfamily family of small GTPases [6,7] consists of GTP binding-dependent molecular switches with diverse cellular functions. The superfamily is classically divided into five subfamilies, namely the Ras, Ran, Rab, Rho and Arf families [8]. Members of these families of small GTPases serve specialized functions in signaling, membrane trafficking, nuclear transport and regulation of cytoskeletal dynamics. Largely synthesized as cytosolic proteins, their activity is regulated by the binding of GTP, facilitated by a myriad of guanine nucleotide exchange factors. With the exception of Ran, family members of Ras, Rab, Rho and Arf are modified by N- or C-terminal attachments of lipid moieties that facilitate their membrane anchorage and function. Ran partitions between the cytoplasm and the nucleus, while the other small GTPases cycle between the cytosol and the plasma membrane, intracellular membranes or the cytoskeleton. No specific Ras superfamily member was known to be specifically associated with the mitochondrion. However, this changes with the discovery of two isoforms of “atypical” Rho GTPases, named mitochondrial Rho (MIRO) [9]. MIRO appears to be fairly conserved in eukaryotes [10] and serves critical roles in mitochondrial morphology [11], inheritance [12,13] and homeostasis [14,15,16]. It is also a key regulator of cytoskeleton-mediated, long-range mitochondrial transport in metazoans [17]. The latter role is of particular importance in the transport of neuronal mitochondria [18,19]. In this review, I shall outline and discuss the known functions of MIRO and the myriad of interacting proteins that it engages in its mitochondria-associated roles.

## 2. MIRO: Gene, Structure and Cellular Interactions

Early work classified MIRO homologues as a novel subgroup of the Rho family GTPases based on sequence homology of its N-terminal GTPase domain with Rho, but subsequent analysis considers these as a distinct, outlying subgroup within the Ras superfamily [6], as MIROs lack an apparent consensus G3 motif and the Rho-specific sequence insert [11]. Human MIRO-1 and MIRO-2 are both 618 amino acids in length, 60% identical to each other and ubiquitously expressed [9]. Their unique domain structure includes two GTPase domains, which flank two calcium-coordinating EF hand domains [20] and a C-terminal transmembrane domain. The N-terminal GTPase domain (but not the C-terminal GTPase domain) has homology to those of Rho family GTPases, but lacking the conserved G12 and Q61 residues, which may indicate a defective GTP hydrolysis activity [9]. Focused phylogenetic analysis indicated that MIRO is present in many eukaryotes, including unicellular yeast, Amoebozoa and multicellular fungi, plants and metazoans. It is however not found in the genome of eukaryotes harboring mitosomes or hydrogenosomes instead of mitochondria and is notably absent in mitochondria-bearing apicomplexans and green algae of the order Mamiellales [10]. There also exist MIRO-like homologues in trypanosomatids and ciliates that lack one of the two GTPase domains [10].

The human paralogues MIRO-1 and MIRO-2 [9,21] and the single *S. cerevisiae* orthologue Gem1p are all localized to the mitochondria, tail-anchored to the outer membrane by the C-terminal transmembrane domain [11]. The importance of Gem1p to yeast mitochondria is demonstrated by the fact that the *gem1Δ* strain grew significantly slower than the wild-type on glycerol minimal media and exhibited distorted mitochondrial morphology and defective mitochondrial distribution [11]. However, Gem1p is apparently not required for mitochondrial division and fusion, and MIRO orthologues are also not required for mitochondrial transport in lower eukaryotes. Metazoan MIRO has critical roles in mitochondrial transport, and this is discussed in the section below. *Miro1* knockout mice could be brought to term, but littermates are cyanotic, have unexpanded lungs and die very shortly after birth [22]. Mouse *Miro2* is therefore of unequal redundancy to *Miro1*. Zebrafish contain three MIRO genes, *rhot1a*, *rhot1b* and *rhot2*, whose products are also shown to be mitochondria-associated [23]. Introduction of antisense morpholinos against either one of the three genes into embryos resulted in no obvious defect, but low-dose triple morphants exhibited a dose-dependent posterior body-axis elongation defect and have smaller heads. High dosages of the combined morpholinos did result in embryonic lethality. MIRO is thus important for development and postnatal life, and is likely so for all metazoans.

MIRO interacts with a number of cellular proteins, and these interactions were indicative of MIRO’s main functions (see Table 1). A yeast two-hybrid-based screen first showed that the *Drosophila* orthologue dMiro interacts with the kinesin adaptor Milton [24]. Subsequent to the early works, MIRO has also been shown to interact with the mammalian Milton homologues OIP106/TRAK1 and GRIF-1/TRAK2 [21,25], as well as the conventional kinesin-1/KIF5 [26,27]. The MIRO/TRAK complex also apparently associates with a myriad of other factors, such as the mitochondrial fusion factors mitofusins 1 and 2 [28], the PTEN-induced putative kinase 1 (PINK1) [14], the neuron-enriched member of the Armcx gene family, Armcx3 [29] and Disrupted In Schizophrenia 1 (DISC1) [30,31]. Pertaining to mitochondrial transport, MIRO also interacts with the retrograde motor dynein [32] and the Hypoxia Upregulated Mitochondrial Movement Regulator (HUMMR) [33,34]. Interestingly, Gem1p is a component of the yeast ER-mitochondria encounter structure (ERMES) tethering complex [35,36]. MIRO is also known to be a substrate of the E3 ubiquitin ligase Parkin [15,16]. A recent report also showed that MIRO interacts with and recruits Centromere protein F (Cenp-F) to the mitochondria to facilitate mitochondrial transport to the periphery of daughter cells after mitosis [37].

In the paragraphs below, I shall discuss MIRO and its interacting partners and their various deciphered functions, in the contexts of both cellular and organismal physiology and pathophysiology.

**Table 1:** A summary of known MIRO interacting partners and their functions.

MIRO interacting protein	Nature of interacting partner	Function/remarks	Reference
Milton (*Drosophila*)	Mitochondrial kinesin motor adaptors	Microtubule-based mitochondrial transport	[24] [21] [25]
OIP106/TRAK1 (mammalian)			
GRIF-1/TRAK2 (mammalian)			
Kinesin 1/KIF5	Kinesin family member of microtubule-based motor proteins	Microtubule-based transport (anterograde)	[26,27]
Dynein	Microtubule-based motor protein	Microtubule-based transport (retrograde)	[32]
Mitofusin 1 and 2	Dynamin-like GTPases	Mitochondrial fusion	[28]
Centromere protein F (Cenp-F)	Centromeric protein	Kinetochore function and chromosome segregation in mitosis	[37]
Disrupted in schizophrenia 1 (DISC1)	Multifunctional scaffold protein	Neural development and multiple signaling pathways, such as Wnt and mTOR; associated with schizophrenia and depression	[30,31]
Hypoxia upregulated mitochondrial movement regulator (HUMMR)	Hypoxia-inducible protein	Axonal mitochondrial transport, particularly in response to hypoxia	[33,34]
PTEN-induced putative kinase 1 (PINK1)	Ser/Thr protein kinase that phosphorylates ubiquitin	Regulator of mitochondrial stress response and mitophagy	[14]
Parkin	E3 ubiquitin ligase	Important component of the ubiquitin-proteasome system of protein degradation; MIRO is a substrate of Parkin	[15,16]

## 3. MIRO’s Role in Intracellular and Intercellular Mitochondria Transport

**Figure 1:**
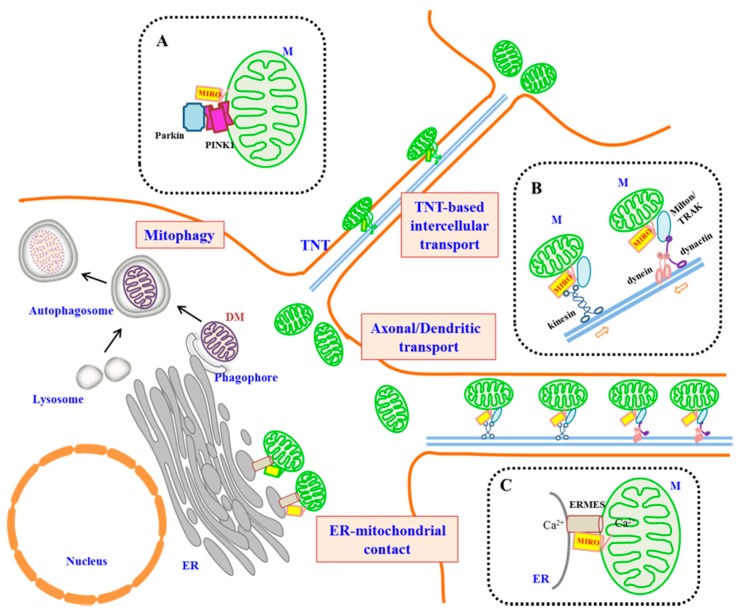
A schematic diagram illustrating the roles of MIRO in metazoans. A generalized cell is shown. Long-distance microtubule-based transport of mitochondria could occur intracellularly (such as in processes of neurons and astrocytes), as well as intercellularly through tunneling nanotubes (TNTs) (not drawn to proportion). Insets (dotted-line boxes) highlight interacting partners of MIRO in three different functional contexts. (**A**) MIRO is the substrate of PINK-1 and Parkin and could be targeted for proteasomal degradation by these proteins. This attenuates mitochondrial mobility and may be a prelude to the mitophagy of damaged mitochondria (DM). (**B**) MIRO complexes with Milton/TRAK and kinesin to mediate anterograde axonal transport of mitochondria, as well as with dynein/dynactin for retrograde transport in axonal and dendritic transport. (**C**) MIRO has been shown to be part of the ER-mitochondrial encounter structure (ERMES) found at ER-mitochondrial contact sites and may have a speculated role in regulating Ca^2+^ exchange.

In neurons of higher metazoans, mitochondrial transport along axons and dendrites is essential for ensuring ATP availability for the energetically-demanding processes at the synapses [38]. Mitochondrial dysfunction, as well as the disruption of mitochondrial transport and distribution underlie a number of peripheral nerve degenerative diseases [39], as well as central nervous system neuronal degeneration, such as those exhibited in Parkinson’s disease [40,41,42]. Axonal mitochondrial transport relies on the microtubule-based motors, namely the kinesin family proteins and dynein [43], and MIRO is associated with both of these classes of motor proteins (see Figure 1). The role for MIRO in anterograde mitochondrial transport in axons was first recognized by an ethyl methanesulfonate (EMS)-based genetic screen in *Drosophila*, where dMiro mutants suffered from locomotion defects and premature death [44]. These mutants have defective mitochondrial transport in both the axon and dendrites. The microtubule-dependent axonal transport is dependent on the function of Milton, a *Drosophila* protein found earlier to be critical for mitochondrial transport [45]. Kinesin was classically known to bind cargo through its light chains. However, Milton recruits kinesin heavy chain (KHC) to mitochondria and associates directly with dMiro [46]. The Milton/KHC/Miro complex is thus a functional complex that works in mitochondrial anterograde transport along microtubules. Two mammalian Milton homologues, OIP106/TRAK1 and GRIF-1/TRAK2 [21,25], were also subsequently shown to associate with mammalian MIRO.

Mitochondrial transport is dependent on cytosolic Ca^2+^ concentration, and the EF hand domains of MIRO appear to have a regulatory role in this regard. Ca^2+^-induced arrest of mitochondrial motility was promoted by MIRO overexpression, and conversely suppressed by either MIRO depletion or the expression of EF hand mutants [47]. As discussed in the section below, MIRO also appears to affect both the motility and fusion-fission dynamics of the mitochondria. There are some uncertainties as to how exactly MIRO modulates kinesin-based mitochondrial transport. In the model of Wang and Schwartz [26], MIRO interacts with kinesin via Milton/TRAK independently of Ca^2+^ (active state). Ca^2+^ binding, on the other hand, promotes the direct interaction of MIRO with the motor domain of kinesin-1, thus dissociating the motor from the microtubule (inactive state) [26]. An alternative model from Kittler’s group suggests that MIRO binds mitochondria directly to kinesin-1/KIF5, and Ca^2+^ binding by MIRO (at micromolar levels) inhibited this interaction [27]. Another possible mode of action involves an axonal mitochondrial docking protein, syntaphilin [48], which is recruited to the axon by neuronal activity [49]. An ”engine switch and brake” model has been proposed by Chen and Sheng [49], whereby MIRO’s Ca^2+^ binding releases kinesin-1/KIF5, thus allowing the latter to interact with syntaphilin, which would restrict further mitochondrial movement.

A few more recently-reported MIRO interacting proteins have now added to the perceived complexity of MIRO’s role in mitochondrial transport. It should be noted that MIRO is required for both anterograde and retrograde axonal transport [50]. MIRO has been shown to associate with the dynein-dynactin complex in lymphocytes [32]; the latter is of course responsible for retrograde axonal transport. MIRO mediates mitochondrial transport into both axons and dendrites, and the latter process is apparently dynein-dependent [51]. Interestingly, the two GTPase domains of dMiro are not equivalent in terms of MIRO function. Expression of an N-terminal GTPase domain mutant (dMiroT25N, dominant-negative mutant with preferential binding to GDP) in the absence of endogenous dMiro caused premature death and developmental arrest at the pupal stage, with mitochondria accumulation in the soma of larval motor and sensory neurons. On the other hand, dMiroT460N did not impair viability and has a much milder phenotype of reduced dynein motility during retrograde mitochondrial transport [51]. HUMMR, which is upregulated by hypoxia, interacts with MIRO, and silencing of HUMMR or its transcriptional regulator hypoxia inducible factor-1α (HIF-1α) during hypoxia diminished axonal mitochondria. HUMMR therefore seems to act in promoting anterograde axonal transport of mitochondria, presumably as a neuronal response to hypoxia [33].

A family of Armadillo (Arm) repeat-containing proteins (encoded by the Armcx gene cluster in the X-chromosome that is unique to the Eutheria clade of mammals) was shown by Soriano and colleagues to be localized to the mitochondria [29]. One of these, Alex3/Armcx3, interacts with the kinesin/Miro/Trak2 complex in a Ca^2+^-dependent manner and appears to affect mitochondrial dynamics and distribution. In a more recent report, the authors also showed that the protein encoded by the *Armc10/SVH* gene (whose retro-transposition gave rise to the Armcx gene cluster) is enriched in neurons and also resides at the mitochondria, interacting with the kinesin/Miro/Trak2 complex [52]. Attesting to its role in mitochondrial dynamics, overexpression of Armc10 prevents amyloid β (Aβ)-induced mitochondrial fragmentation [52]. Another interesting protein that was recently shown to interact with TRAK1 and MIRO is Disrupted In Schizophrenia 1 (DISC1), a key susceptibility factor for psychiatric disorders [53]. DISC1 has been shown to regulate neuronal mitochondrial trafficking [54]. It apparently acts to promote anterograde mitochondrial transport, and this ability is lost in a putatively disease causing human DISC1 sequence variant, 37W [30]. The DISC1-Boymaw fusion protein (arising from a schizophrenia-associated chromosomal translocation, which interrupts DISC1 in a Scottish pedigree [55]) was shown to localize to the mitochondria, disrupts mitochondrial dynamics [31,56] and affects dendritic development. Pathological forms of DISC1 may therefore act by disrupting neuronal mitochondrial dynamics via its interaction with the MIRO/TRAK complex.

Not only is MIRO involved in mitochondrial transport to the remote peripheral regions of specialized cell types, it was also shown to mediate movement of mitochondria between cells [57]. A particularly interesting mode of intercellular communication occurs via physical wiring between the cells through actin-based structures known as tunneling nanotubes (TNTs) [58,59]. Mesenchymal stem cells (MSC), either co-cultured *in vitro* or introduced *in vivo*, could often aid the survival and recovery of damaged or injured recipient/host cells [60]. This could occur by a range of mechanisms, including paracrine secretion of soluble factors or those carried in microvesicles [61]. TNTs are increasingly known to mediate intercellular communications of this nature [62], and some TNTs bearing microtubules are capable of transporting not just small molecules, but organelles, such as lipid droplets [63], lysosomes [64,65] and mitochondria [66,67]. This intercellular transfer of mitochondria could apparently aid cellular regeneration, presumably by functional replacement of damaged or diseased mitochondria in recipient cells [68,69,70]. A recent report has now shown that MIRO regulates this intercellular transfer of mitochondria between MSCs and airway epithelial cells in a mouse model of airway injury and allergic airway inflammation [71]. Overexpression of MIRO in MSCs resulted in enhanced mitochondrial transfer to the recipient epithelial cells and reversed airway hyper-responsiveness to allergen-induced asthma. These results suggest that MIRO manipulation could enhance the therapeutic potential of MSCs.

## 4. MIRO’s Role in Mitochondria Homeostasis

Other than transport and distribution, mitochondria are dynamic organelles that undergo frequent changes in morphology, as well as fusion and fission [72,73]. Although MIRO was not initially thought to be acting in the classical pathways that regulate mitochondrial fusion/fission, yeast *gem1*Δ mutants nonetheless exhibited distorted mitochondrial morphology [11]. In rat cardiomyocytes (H9c2 cells), overexpression of MIRO1 induced mitochondrial thread formation and condensation. On the other hand, dominant-negative MIRO constructs and silencing of MIRO caused mitochondrial fragmentation [47]. The effects of MIRO on mitochondrial morphology seem to involve the suppression and activation of a key regulator of mitochondrial division, the Dynamin-related protein 1 (Drp1) [74]. Overexpression of wild-type MIRO1 or MIRO2 in primary neurons moderately increased mitochondrial length, and this increase is heightened by the expression of the EF hand mutants of MIRO. MIRO’s Ca^2+^ binding capacity could thus modulate its influence on mitochondrial morphology [47]. MIRO’s interaction with mitofusins 1 and 2 [28] may also influence mitochondrial fusion, but the detailed mechanism in this regard is not clear.

The Ser/Thr kinase PINK1 and the E3 ubiquitin ligase Parkin act in a cooperative manner in sensing the health and functionality of the mitochondria and label damaged mitochondria by ubiquitination for mitophagy [75]. The fact that MIRO interacts with PINK1 [14] and Parkin [15,16] suggests that it is one of the targets of the PINK1-Parkin system and would thus play a role in mitochondrial turnover. Indeed, it was shown that PINK1 phosphorylates MIRO [76], and MIRO, being a substrate of Parkin E3 ligase activity [15], could be ubiquitinated and targeted for proteasomal degradation when PINK-1 and Parkin associate with damaged mitochondria. MIRO removal limits mitochondrial movement and may serve the purpose of their confinement prior to destruction by mitophagy [76]. In HeLa cells, loss of MIRO resulted in the perinuclear clustering of mitochondria and facilitated mitophagy [77]. In *Drosophila*, PINK1 phosphorylation-resistant mutants of dMIRO in a dMIRO-null background exhibited increased mitochondrial transport and synaptic over-growth at neuromuscular junctions, as well as dopaminergic neuron degeneration in the brains of adult. These partially resembled the phenotypes of PINK1 null flies, and PINK1/Parkin-mediated degradation of MIRO may thus be beneficial under certain diseased conditions.

## 5. MIRO’s Roles in Plants and Lower Eukaryotes

The *Arabidopsis* genome harbors three MIRO paralogues, *MIROs-1–3* [78]. *MIRO-1* and *MIRO-2* are ubiquitous, but *MIRO-3* is specifically expressed in the endosperm [17]. While transfer (T)-DNA insertional mutation of *Arabidopsis MIRO-1* resulted in lethality during embryogenesis, insertional mutation of *MIRO-2* had little effect [79]. Phylogenetic analysis revealed that *MIRO-1* and *MIRO-2* in dicot plants cluster in two separate groups due to a gene or genome duplication event [80], but the above results indicate that they are unequally redundant. Loss of *MIRO-1* causes clear impairment in pollen germination and pollen tube growth. Mitochondria in the *MIRO-1* mutant pollen exhibited abnormal morphology and intracellular distribution, but the mutation does not seem to affect actin-dependent mitochondrial motility [78]. Loss of *MIRO-2* in the heterozygous *miro1+/-* background enhanced the pollen tube growth defects and impaired or delayed the fusion of polar nuclei [80]. A closer examination of mitochondria in eggs and early-stage embryos showed that these are abnormally enlarged in the *MIRO-1* mutant, and the apical cell of a two-celled embryo contained a reduced number of mitochondria compared to the wild-type [79]. The latter would indicate some defect in mitochondrial inheritance. However, mitochondria in the *MIRO-1* mutant continue to undergo actin-dependent cytoplasmic streaming. Coupled to a lack of a Milton/TRAK-like protein in *Arabidopsis*, MIRO-modulation of mitochondrial transport that is equivalent to that occurring in metazoan animals is apparently absent in plants.

The MIRO orthologue of the budding yeast *S. cerevisiae* is important for the maintenance of tubular mitochondrial morphology and also mitochondrial inheritance in daughter buds [11]. In *gem1*Δ mutants, cells contain large, globular mitochondria, and small-budded (but not large-budded) cells exhibited an inheritance delay. *gem1*Δ also exhibits synthetic growth defects with other genes that are known to be play roles in mitochondrial distribution, such as that encoding mitochondrial MYO2 receptor-related protein 1 (MMR1p). The *gem1Δ mmr1Δ* double mutant exhibited a more severe inheritance defect in small-budded cells than single mutants [81]. In fact, it was shown that mutating either one of the two GTPase domains is sufficient to affect inheritance [12]. Although Ca^2+^ binding by the EF hand domains did not appear to be directly involved in mitochondrial inheritance, a functional N-terminal EF hand was apparently critical for the stable expression of Gem1p.

Mitochondria could be associated directly with membranes of the ER via the ER-mitochondria contact sites [82,83]. A physical link, in the form of the ER-Mitochondria Encounter Structure (ERMES) tethering complex, was initially found to play a role in phospholipid exchange between the two compartments and various aspects of mitochondrial function [84]. Gem1p was shown to be a component of ERMES and appears to regulate its numbers and sizes [35]. *In vitro* molecular dissections indicated that the first GTPase domain and the first of the EF hands are required for Gem1p’s ERMES association, whereas the second GTPase domain is required for phospholipid exchange. In this regard, it should also be noted that mitofusin 2, which interacts with MIRO-TRAK [28], has been shown to mediate ER-mitochondrial contact [85]. However, ER-mitochondria contacts tend to also be sites of ER-associated mitochondrial division, and another report has indicated that Gem1p actually acts by antagonizing the ER-mitochondrial contacts to aid mitochondrial segregation [36]. Furthermore, a report that argued that ERMES and Gem1p have no direct roles in ER-mitochondrial transport of phosphatidylserine also found the ERMES complexes to be stable, long-lived structures, the existence of which is not dependent on Gem1p [22]. ER-mitochondrial contact sites are now known to be major sites of autophagosome formation [86,87] and, in this regard, would be important for mitophagy. The role of Gem1p and the mammalian MIROs in ER-mitochondrial contact sites clearly deserves further investigations.

The slime mold *Dictyostelium discoideum* has a single MIRO gene, *gemA* [10]. Disruption of *gemA* resulted in growth impairment, but no visible alterations to mitochondrial size and morphology and no significant changes to mitochondrial function except for an increase in oxygen consumption. Mitochondrial distribution in *D. discoideum* is dependent on microtubules, but there is no appreciable difference between mitochondrial distribution in *gemA* and wild-type cells. The role of MIRO has not yet been examined in other lower eukaryotes, but despite it being evolutionarily conserved, its role in microtubule-mediated mitochondria transport is postulated to have evolved only in metazoans [17].

## 6. MIRO and Diseases

As a key regulator of mitochondrial transport and dynamics, MIRO would be expected to be somewhat involved or implicated in diseases associated with defects in mitochondrial movement and function, particularly neurodevelopmental and neurodegenerative disorders. In general, mitochondrial defects have been extensively associated with Parkinson’s disease (PD) and Alzheimer’s disease (AD) [88]. As discussed above, the PD-associated [89] proteins PINK-1 and Parkin interact with MIRO and affects its degradation and mitochondrial motility [14,15,76,77,90]. A direct association between MIRO and AD has not yet been shown, but MIRO levels are known to be downregulated in the presenilin 1 E280A mutation that is associated with familial AD [91]. Overexpression of the kinesin/Miro/Trak2 interactor Armc10 could prevent amyloid β (Aβ)-induced mitochondrial fragmentation [52]. Changes in another MIRO-TRAK interactor, mitofusin 2 [28], has also been recently implicated in tauopathy and AD-associated pathology [92,93]. Loss of mitochondria by dMIRO silencing in the axons of transgenic *Drosophila* expressing human tau have been shown to promote tau phosphorylation and AD pathology [94]. Dominantly-inherited point mutations in mitofusin 2 are known to underlie peripheral axon degeneration in human Charcot–Marie–Tooth (CMT) disease (CMT type 2A) [95]. Mutations in the *Drosophila* orthologue of mitofusin, mitochondrial assembly regulatory factor (Marf), have also been shown to be required for mitochondrial transport in long axons, and loss of Marf resulted in a depletion of mitochondria in neuromuscular junctions [96]. Details as to how MIRO and mitofusins function together in neuronal physiology and pathology await future investigations.

Another neurodegenerative disease with defined connections with MIRO is Amyotrophic Lateral Sclerosis (ALS). MIRO1 was significantly reduced in the spinal cord tissue of ALS patients, as well as transgenic mice expressing familial ALS-associated superoxide dismutase 1 (SOD1) G93A or TAR DNA binding protein-43 (TDP-43) M337V mutant genes [97]. Mutations in the vesicle-associated membrane protein-associated protein B (VAPB), such as VAPBP56S, cause a familial form of ALS (type 8). Expression of the VAPBP56S mutant in rat cortical neurons disrupts anterograde axonal mitochondrial transport, apparently by increasing resting Ca^2+^, which affected MIRO’s modulation of mitochondrial transport [98]. Interestingly, overexpression of a Ca^2+^-insensitive, EF hand mutant of MIRO could rescue defective mitochondrial axonal transport caused by the VAPBP56S mutant.

Cellular regulators of MIRO’s GTP binding status and GTPase activity are not well known. However, MIRO is apparently the target of a pathogen effector with GTPase-activating protein (GAP) mimicking activity. *Vibrio cholerae* type 3 secretion system effector VopE, which localizes to mitochondria during infection, binds to the GTPase domain and could act as a GAP for MIRO1 and MIRO2 [99]. The mitochondrial antiviral-signaling protein (MAVS) plays an important role in NF-κB and type I interferon signaling [100]. VopE appears to inhibit MAVS-mediated IκB kinase activation. As MAVS does not appear to directly interact with VopE-MIRO, VopE’s action on MAVS could be indirect and likely through the alteration of MIRO-mediated mitochondrial dynamics.

## 7. Epilogue

In the paragraphs above, I have discussed the roles of MIRO and its interactors in mitochondrial homeostasis and transport (see Figure 1). Much remains to be learned about MIRO’s mechanism of function. The relative importance of the MIRO paralogues 1 and 2 in key cellular processes and during development of mammals is not particularly clear. Little is known about how the GTPase activities of MIRO’s GTPase domains is regulated by upstream regulators, such as GDP-GTP exchange factors and GAPs. The actual mechanism of how the MIRO/TRAK/kinesin complex works in mitochondrial movement along microtubules has remained unresolved, and little is known about how MIRO works with dynein-dynactin. While it is clear that MIRO is a PINK1 and Parkin substrate, the cellular condition and contexts in which these enzymes are able to engage MIRO are unclear. It is also not known if MIRO simply has a passive role in mitochondrial turnover or could actively contribute to mitophagy. MIRO’s association with ERMES in yeast remains controversial and its role in ER-mitochondrial contact sites in mammalian cells has not been explored. There is a tantalizing connection between MIRO’s link with PINK1/Parkin modulated mitophagy and ER-mitochondrial contact sites being locations of autophagosome origin, but the detailed implications of these links are not yet known.

MIRO’s roles in mitochondrial dynamics have also provided tantalizing links with diseased states, particularly CNS and peripheral neurodegeneration. The connections are, however, rather tentative for most cases at the moment. Future work may reveal further insights into the pathophysiological roles of MIRO in various disease models and may shed more light onto those associated with sporadic and idiopathic forms of neurodegeneration with defects in mitochondrial dynamics. The recent finding that increased MIRO expression could enhance mitochondrial transfer from MSCs to recipient cells is highly interesting and could potentially be exploited as a therapeutic strategy in regenerative medicine.

## Abbreviations

The following abbreviations are used in this manuscript:

ER: endoplasmic reticulum lysosome
ERMES: ER-mitochondrial encounter structure
M: mitochondrion
TNT: tunneling nanotube
